# Age distribution of Antarctic Bottom Water off Cape Darnley, East Antarctica, estimated using chlorofluorocarbon and sulfur hexafluoride

**DOI:** 10.1038/s41598-022-12109-4

**Published:** 2022-05-19

**Authors:** Yoshihiko Ohashi, Michiyo Yamamoto-Kawai, Kazuya Kusahara, Ken’ichi Sasaki, Kay I. Ohshima

**Affiliations:** 1 Department of Ocean Sciences, Tokyo University of Marine Science and Technology, Tokyo, 108-8477 Japan; 2 Research Center for Environmental Modeling and Application, Research Institute for Global Change, Japan Agency for Marine-Earth Science and Technology, Yokohama, 236-0001 Japan; 3 Mutsu Institute for Oceanography, Research Institute for Global Change, Japan Agency for Marine-Earth Science and Technology, Mutsu, 035-0022 Japan; 4 Institute of Low Temperature Science, Hokkaido University, Sapporo, 060-0819 Japan; 5 Arctic Research Center, Hokkaido University, Sapporo, 001-0021 Japan

**Keywords:** Ocean sciences, Marine chemistry, Physical oceanography

## Abstract

Chlorofluorocarbon (CFC) and sulfur hexafluoride (SF_6_) were used to investigate the timescale of Antarctic Bottom Water (AABW) that spreads off Cape Darnley (CD) in East Antarctica. The age of the AABW was estimated based on the observed SF_6_/CFC-12 ratio while taking into account tracer dilution by Lower Circumpolar Deep Water. Along the western canyons off CD and the ~ 3000 to 3500 m isobaths, the bottom water age was < 5 years, reflecting the spread of newly formed CD Bottom Water. Higher ages of ~ 8 years obtained for areas east of CD and > 20 years in the northwestern offshore region indicate inflows of AABW through the Princess Elizabeth Trough and Weddell Sea Deep Water, respectively. This study determined the age distribution in the region off CD, where three different types of AABW spread.

## Introduction

On the Antarctic continental shelf, cold Dense Shelf Water (DSW) forms through cooling and brine rejection during ice formation in coastal polynyas. With strong buoyancy loss, the DSW flows down the continental slope and mixes with ambient Circumpolar Deep Water (CDW) to produce Antarctic Bottom Water (AABW)^1^. AABW continues to mix with overlying and adjacent waters as it is advected. The production of AABW drives global thermohaline circulation, delivering oxygen and carbon to the global abyssal ocean (e.g., refs.^2–5^). Thus, a quantitative assessment of the production and spread of AABW is critical for understanding global ocean circulation and climate.

The Weddell and Ross Seas, and the Adélie Land coast are known as the three major regions of AABW production^6–9^. Recently, ref.^10^ found that AABW production off Cape Darnley (CD) occurs due to intense sea ice production^11^. The main source of this AABW (Cape Darnley Bottom Water; CDBW) is DSW that formed in CD polynya^10,12^, with a contribution of DSW outflow from Prydz Bay^13^. The CDBW mainly descends the canyons in a northwestward direction^10,12,14^. In addition to the CDBW, two other types of AABW are present in the region off CD. From the east, warmer and more saline AABW (a mixture of Ross Sea Bottom Water and Adélie Land Bottom Water) flows into the region through the Princess Elizabeth Trough (PET)^3,15^. From the west, AABW produced in the Weddell Sea (Weddell Sea Deep Water; WSDW) is transported into the region by the Weddell Gyre^16,17^. Although the regions in which each AABW type is produced and their transport pathways are roughly known, the timescale of AABW transport is not clearly understood.

Anthropogenic gases such as chlorofluorocarbons (CFCs) and sulfur hexafluoride (SF_6_) have been used as transient tracers to detect newly ventilated water masses and to quantitatively understand water mass spreading pathways and their timescales. Their equilibrium concentrations in seawater are determined by their atmospheric concentrations and solubility at the surface. In the real ocean, concentrations in surface water are also affected by physical processes, such as the rate and degree of gas exchange, which are altered by cooling, warming, vertical mixing, as well sea ice cover in polar oceans. In the interior of the ocean, their concentrations change only by mixing with other water masses, as they are chemically and biologically inert (e.g., ref.^18^). As atmospheric time histories for these gases are well established^19^, their concentrations in a water parcel can be used to estimate the year in which the water was last in contact with the atmosphere. In the Weddell and Ross Seas, previous studies have estimated the timescale of water mass spread using the partial pressure of CFC (pCFC) age or its ratio (pCFC-11/pCFC-12) age (e.g., refs.^20,21^). The age means the time elapsed since the water was in contact with the atmosphere. The pCFC age is determined by comparing the pCFC of the water (defined as the observed tracer concentration divided by the solubility function^22,23^) with the atmospheric time history (e.g., ref.^19^). The pCFC ratio age is also used in a similar way as the pCFC age. In the region off CD, the CFC maxima in the bottom water suggest that the AABW formation occurs around 60°–70°E^24^. In the downstream Weddell Sea, an intrusion of high CFC water from the east suggested inflow of new AABW from the CD region^25,26^. However, water mass ages and the transport timescale for CDBW have not been investigated. The AABW found off CD should exhibit a broad range of spreading timescales, from a few years to decades due to inflows of AABW from different source regions. However, because the atmospheric pCFC and its ratio reached a maximum during the 1980s–2000s, after which their temporal changes are small^18,19,21^, it is difficult to uniquely determine the age in the region off CD by applying these previous methods. Although the partial pressure of SF_6_ (pSF_6_) is still increasing in the atmosphere, the pSF_6_ age (as well as the pCFC age) cannot realistically estimate the mean age when waters of different ages mix. As the pSF_6_/pCFC ratio is still increasing in the atmosphere (e.g., ref.^18^), their ratio age might be able to estimate the AABW age, especially if the CDW is essentially CFC and SF_6_-free. Other studies of CFC and SF_6_ over the past couple decades have used the concept of Transit Time Distribution (TTD) to estimate the water mass age (e.g., ref.^27^). The 1-D TTD, which is a solution of the 1-D advection–diffusion equation^28^, is not applicable in the Southern Ocean where distinct water masses are mixing, especially when one endmember has essentially no tracers (e.g., ref.^29^). Reference^30^ found that the other forms of simulated TTD might be applicable in the Southern Ocean. However, the water mass age estimated by the TTD method represents the mean age of a mixture of different water masses, not the age since the mixture is formed. In case of AABW, mean age can be already old at the time of formation, because it is formed by the mixing of old CDW with new DSW. Therefore, mean age is not appropriate for application in studying the timescale of AABW spread.

**Figure 1:**
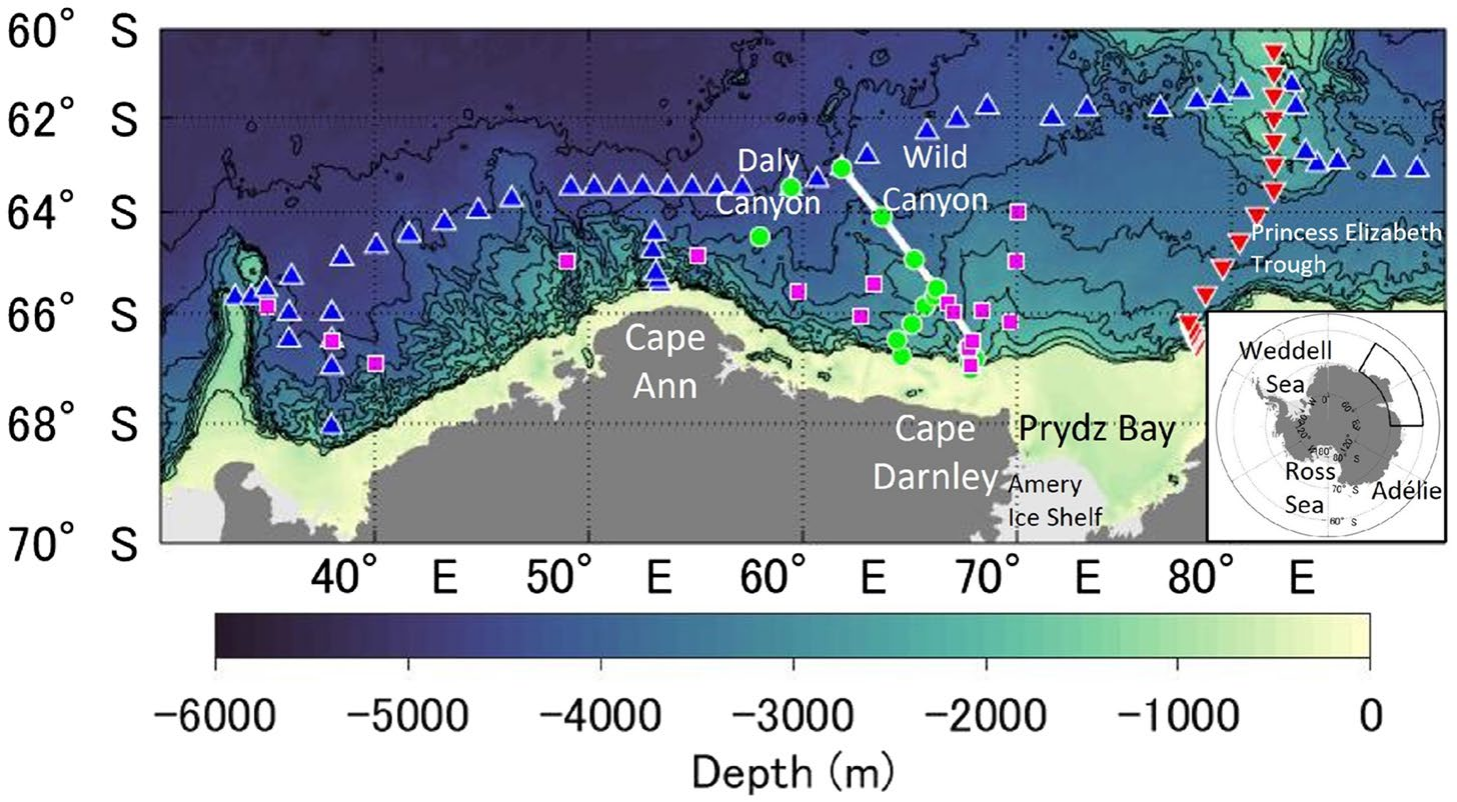
Study area and locations of the observation sites. The inset shows the location of the study area. Colored symbols denote the observation sites in 2013 (blue triangles; MR12-05), 2016 (red inverted triangles; WHP I08S), 2019 (green circles; KH19-01), and 2020 (magenta squares; KH20-01). Data collected from the sites along the white line are shown in Figs. 2 and 8. The ocean bathymetry is based on the RTopo-2 dataset^59^. The figure was generated using MATLAB (version R2021a; https://www.mathworks.com/products/matlab.html) and the M_Map toolbox (http://www.eoas.ubc.ca/~rich/map.html).

In this study, in order to quantitatively determine the pathway and timescale of AABW spread in the region off CD, we observed CFC-12 and SF_6_ (Fig. 1; see “Methods” for details). Distributions of pCFC-12 and pSF_6_ show the spread of newly ventilated CDBW from its formation region to the offshore region. Based on an analysis of the observed pCFC-12 and pSF_6_, we propose a method using the pSF_6_/pCFC-12 ratio while taking into account tracer dilution by Lower CDW (LCDW) for estimating the age of the AABW. Using the method, we present the distribution of AABW ages in the study area. Numerical modeling of CFC-12 and SF_6_ was also performed to investigate the applicability of the estimation method.

## Results and discussion

### Water mass distributions along Wild Canyon

**Figure 2:**
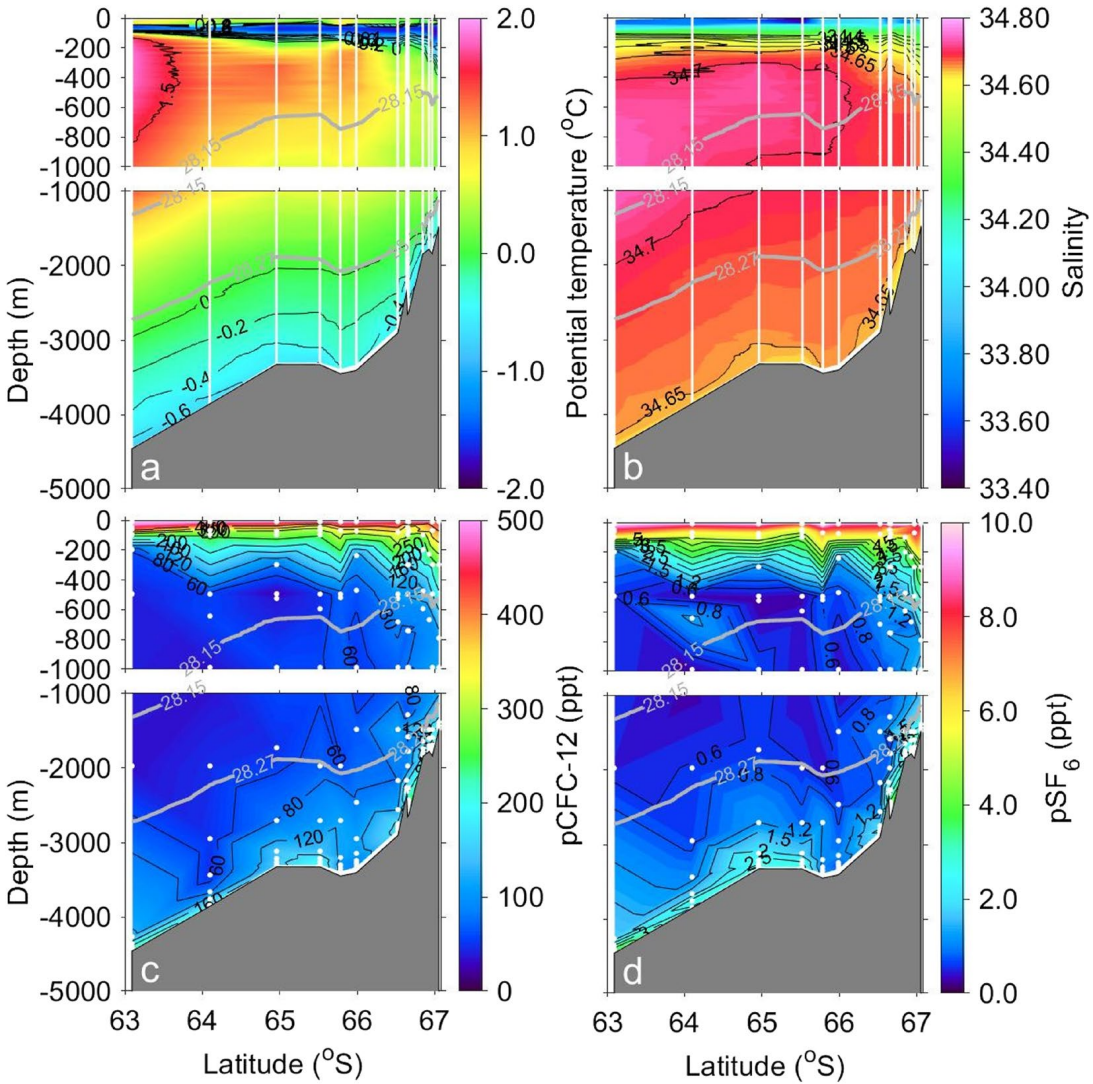
Contour plots of (**a**) potential temperature, (**b**) salinity, (**c**) pCFC-12, and (**d**) pSF_6_ along Wild Canyon (white line in Fig. 1). Data were obtained during cruises of KH19-01 and KH20-01. Gray horizontal lines indicate neutral densities of 28.15 kg m^−3^ and 28.27 kg m^−3^. The figure was generated using MATLAB (version R2021a; https://www.mathworks.com/products/matlab.html).

Vertical sections of water properties along Wild Canyon in the west of CD are shown in Fig. 2. To identify the water mass properties, we used the neutral density (*γ*^n^: kg m^−3^)^31^ and potential temperature referring to refs.^32–34^. Relatively warm water occupied the region from the surface to a depth of 50 m (*θ* > 0.0 °C; Fig. 2a). Below the warm surface water, the potential temperature decreased with depth to a minimum (*θ* =  − 1.6 °C; Fig. 2a). This temperature minimum is known as winter water, which is the remnant of the previous winter’s mixed layer (*θ* <  − 1.5 °C; e.g., refs.^35,36^). From the surface to ~ 100 to 200 m, the combined warm surface water and winter water are known as Antarctic Surface Water (AASW; e.g., ref.^37^). In the AASW layer, low salinity and high pCFC-12 and pSF_6_ were observed (*S* < 34.60, pCFC-12 > 250 ppt, pSF_6_ > 4.0 ppt; Fig. 2b–d). pCFC-12 and pSF_6_ values were the highest among water masses but lower than the atmospheric equilibrium concentrations. The mean pCFC-12 and pSF_6_ in warm surface water were 94% and 87% of the atmospheric concentrations, respectively, while those in cold winter water were 66% and 63% of the atmospheric concentrations, respectively.

Warm saline water below the AASW originates from the CDW. CDW is divided into the Upper CDW (UCDW; *γ*^n^ ≤ 28.15 kg m^−3^) and the LCDW (28.15 kg m^−3^ < *γ*^n^ ≤ 28.27 kg m^−3^). The UCDW/LCDW reached ~ 400 m/ ~ 1200 m depth at the coastal station (~ 67°S), while reaching ~ 1300 m/ ~ 2700 m depth at the farthest offshore station (~ 63°S). Although the potential temperature decreased with depth below ~ 500 m, the temperature was still greater than 0.0 °C at the deeper end of the LCDW (Fig. 2a). Salinity exhibited a maximum around the boundary of UCDW and LCDW (*S* > 34.70; Fig. 2b). Although the pCFC-12 and pSF_6_ were relatively low in the whole CDW, minima were observed in the LCDW at most of the stations (pCFC-12 < 60 ppt, pSF_6_ < 0.6 ppt; Fig. 2c,d). In addition, from the offshore region to the coast, the potential temperature and salinity of UCDW/LCDW decreased, while the pCFC-12 and pSF_6_ increased.

The AABW (*γ*^n^ > 28.27 kg m^−3^) near the bottom was particularly cold, fresh, and high in pCFC-12 and pSF_6_ (*θ* <  − 0.4 °C, *S* < 34.64, pCFC-12 > 120 ppt, pSF_6_ > 1.5 ppt; Fig. 2a–d), except around ~ 66°S where warm, saline, low pCFC-12 and low pSF_6_ conditions were observed.

### AABW properties near the bottom

**Figure 3:**
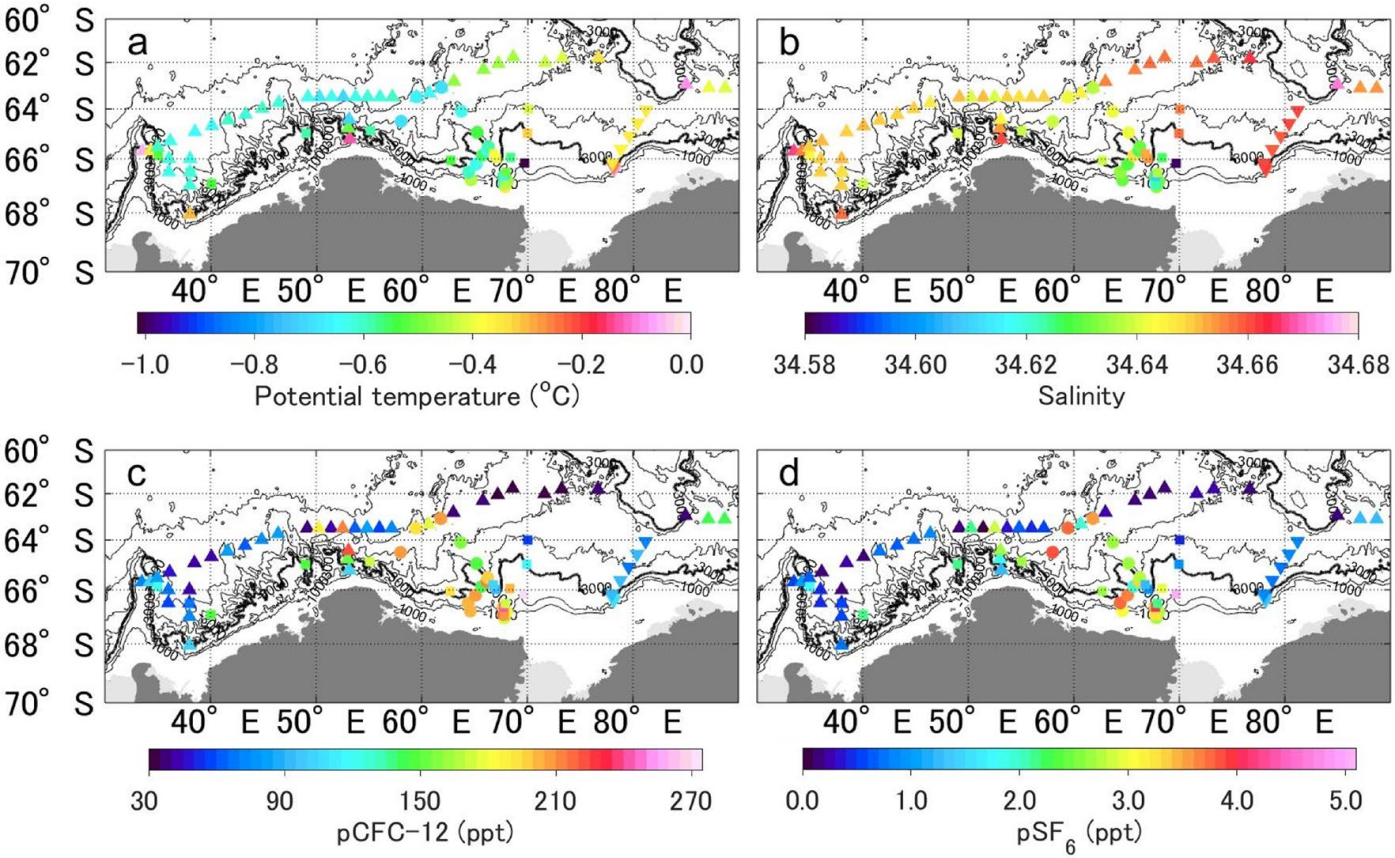
Spatial distributions of bottom (**a**) potential temperature, (**b**) salinity, (**c**) pCFC-12, and (**d**) pSF_6_ of the AABW (neutral density: *γ*^n^ > 28.27 kg m^−3^) (mean of observations within 100 m of the bottom). The symbols used are the same as in Fig. 1. Note that pSF_6_ in the atmosphere increased by 20% between 2013 and 2020. Therefore, the distribution of pSF_6_ in AABW can vary considerably among observation years. Black contour lines represent isobaths at 1000 m intervals from 1000 to 3000 m and at 500 m intervals below 3000 m. The 3000 m isobath is indicated by the thick black line. The figure was generated using MATLAB (version R2021a; https://www.mathworks.com/products/matlab.html) and the M_Map toolbox (http://www.eoas.ubc.ca/~rich/map.html).

Figure 3 shows the spatial distributions of the AABW properties near the bottom (mean of observations within 100 m of the bottom) in the study area. From off CD to the northwest and along the 3000 m and 3500 m isobaths, AABW had lower salinity and higher pCFC-12 and pSF_6_ than the other stations (*S* < 34.64, pCFC-12 > 120 ppt, pSF_6_ > 1.5 ppt; Fig. 3b–d), except around ~ 66°S in Wild Canyon. In the region east of Wild Canyon, the AABW was characterized by higher temperature, higher salinity, lower pCFC-12, and lower pSF_6_ (*θ* >  − 0.4 °C, *S* > 34.65, pCFC-12 < 120 ppt, pSF_6_ < 1.5 ppt; Fig. 3a–d) than in the region west of Wild Canyon. In the northwestern part of the study area, AABW with lower temperature, lower pCFC-12, and lower pSF_6_ was found (*θ* = up to − 0.6 °C, pCFC-12 < 100 ppt, pSF_6_ < 1.0 ppt; Fig. 3a,c,d).

### Estimation of the age of the AABW

**Figure 4:**
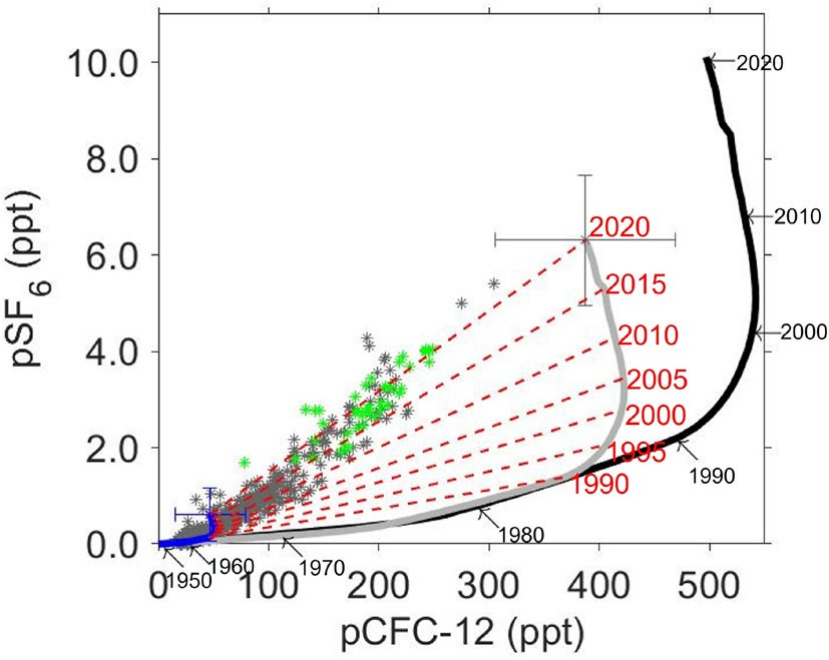
Relationship between observed pCFC-12 and pSF_6_ in AABW. Green and dark gray asterisks indicate new AABWs along Wild Canyon (neutral density: *γ*^n^ > 28.27 kg m^−3^, *θ* <  − 0.4 °C)^10^ and the other AABWs (*γ*^n^ > 28.27 kg m^−3^), respectively. Black line and black numbers indicate the year-to-year variations in atmospheric partial pressure and the year, respectively. Blue and gray lines indicate the year-to-year variations in LCDW and DSW, respectively. Blue and gray crosses show the averages and standard deviations of the estimated LCDW and DSW in 2020, respectively. Red dashed lines and red numbers indicate the estimated year-to-year linear relationship of AABW and the year, respectively.

**Figure 5:**
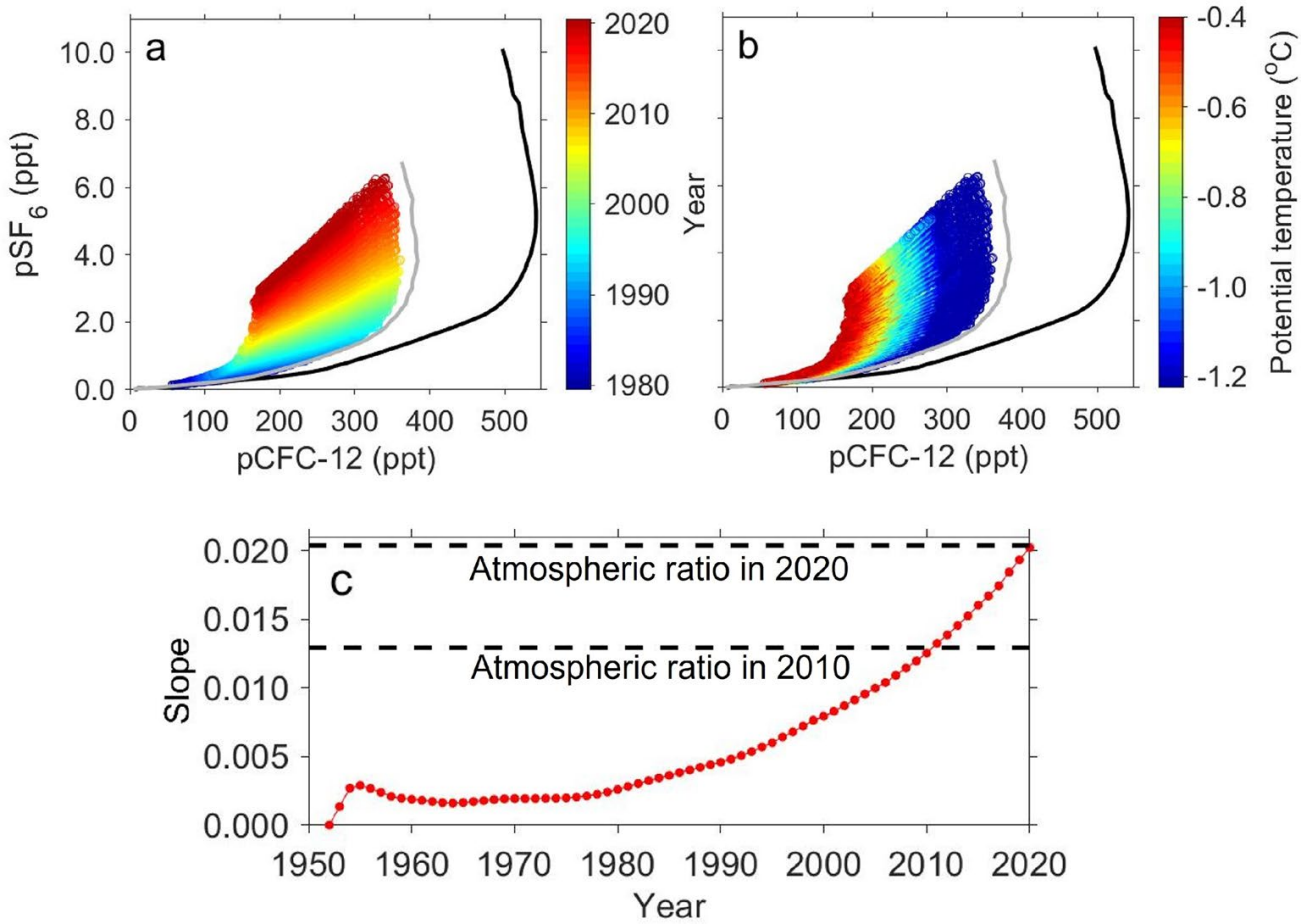
Relationship between simulated pCFC-12 and pSF_6_ in new AABW (in the region near the Cape Darnley polynya; 63°–67°S, 60°–68°E, neutral density: *γ*^n^ > 28.27 kg m^−3^, *θ* <  − 0.4 °C)^10^. Data are averaged over January–December for each year. In (**a,b**), data from 1980 to 2020 are used. Black and gray lines indicate the year-to-year variations in atmospheric partial pressure and winter (June–August) mean DSW (surface values at Cape Darnley polynya: ≥ 67°S, 65°–70°E), respectively. Colors denote (**a**) year and (**b**) potential temperature. (**c**) Year-to-year variations in the slope of the approximately straight lines based on the relationship between simulated pCFC-12 and pSF_6_ in new AABW (red). Dashed black lines indicate the atmospheric pSF_6_/pCFC-12 ratio in 2010 and 2020.

To understand the timescale of the spread of AABW, we estimated the water mass ages of the AABW from the observed pSF_6_/pCFC-12 ratio. Here, we defined the AABW age as the year when the AABW was formed due to the mixing of LCDW and DSW (e.g., refs.^38,39^), instead of the mean age of the mixture. In order to estimate the ages from pCFC-12 and pSF_6_, an understanding of how the concentrations of these gases in AABW change from year to year is required. The observed pCFC-12 and pSF_6_ in new AABW (green asterisks in Fig. 4)^10^ were distributed linearly between old water (almost zero pCFC-12 and pSF_6_) and new water (high pCFC-12 and pSF_6_). This indicates that pCFC-12 and pSF_6_ in the AABW are results from the mixing of two endmembers (old LCDW and recently ventilated DSW). This linear relationship between pCFC-12 and pSF_6_ in AABW should vary with time, reflecting changes in pCFC-12 and pSF_6_ in the DSW, which are proportional to atmospheric concentrations. Accordingly, using the relationship between pCFC-12 and pSF_6_, the year when the DSW contained in the AABW was in contact with the atmosphere, and thus the AABW age could be estimated. Now, we need to know how pCFC-12 and pSF_6_ in the DSW, as well as in the LCDW as another endmember, change from year to year. First, the observed pCFC-12 and pSF_6_ of the LCDW in the study area (28.15 kg m^−3^ < *γ*^n^ ≤ 28.27 kg m^−3^) were compared with the atmospheric partial pressures during the observed year to obtain mean saturations of 9 ± 6% (standard deviation: SD) and 6 ± 6%, respectively. The saturation values were close to those over the circumpolar Southern Ocean (> 55°S, pCFC-12: 6%, pSF_6_: 4%; calculated from Global Ocean Data Analysis Project [GLODAP] v2.2020 dataset; ref.^40^). The observed pCFC-12 and pSF_6_ of the LCDW, although they were relatively low throughout the study area, increased from the offshore region to the coast (e.g., Fig. 2c,d). This suggests that the concentrations of these gases in the LCDW increased due to mixing with newly ventilated shelf water. Observations in the Weddell, Ross, Somov, and Lazarev Seas (ref.^41^), also show that LCDW rises at the continental slope and mixes with the shelf water to increase the pCFC-12 and pSF_6_ in LCDW. Therefore, the pCFC-12 and pSF_6_ of the LCDW likely vary in response to the temporal evolution of the concentrations of these gases in shelf water that is in contact with the atmosphere, and reflect the temporal variations in the atmospheric partial pressures. For this reason, we assumed that the pCFC-12 and pSF_6_ in the LCDW (blue line in Fig. 4) varied in proportion to the atmospheric partial pressures (ref.^19^ for years before 2015 and data obtained from NOAA Global Monitoring Laboratory for years after 2016: https://gml.noaa.gov/hats/combined/CFC12.html and https://gml.noaa.gov/hats/combined/SF6.html; black lines in Figs. 4, 5a,b). For the DSW, as it forms during the winter, no direct observations of pCFC-12 and pSF_6_ are available. Therefore, we determined the properties of the DSW from those of the new AABW and LCDW. From the potential temperature, the mixing ratio of LCDW (*θ* = 0.4 °C) and DSW (*θ* =  − 1.9 °C) was estimated for each observed new AABW. We assumed that the pCFC-12 and pSF_6_ of each new AABW was a mixture of those in the LCDW (*θ* = 0.4 °C, pCFC-12 = 9%, pSF_6_ = 6%) and the DSW at the estimated mixing ratio. Then, the pCFC-12 and pSF_6_ of the DSW were estimated to be 78 ± 16% and 62 ± 13% of the atmospheric partial pressure, respectively. Previous studies have also reported higher saturation for pCFC-12 than for pSF_6_ in surface waters (12% difference in North Atlantic; ref.^42^, 8% difference in Arctic Ocean; ref.^43^). As suggested by ref.^42^, the differences in saturation between pCFC-12 and pSF_6_ can reflect differences in atmospheric time histories or piston velocities between pSF_6_ and pCFC-12. In addition, the differences could be attributed to the seasonal entrainment of lower tracer subsurface waters^44^. The estimated pCFC-12 and pSF_6_ for the DSW were relatively close to those observed in winter water, which is considered to be a proxy for the previous winter’s mixed layer in the study area (*θ* <  − 1.5 °C, pCFC-12: 67 ± 11%, pSF_6_: 64 ± 12%). In other AABW formation regions, the mean saturations of CFC-12 and SF_6_ calculated from the GLODAPv2.2020 dataset were 67 ± 0% and 63 ± 1%, respectively, in DSW in the Adélie Land coast region (n = 2); 75 ± 5% and 69 ± 6%, respectively, in winter water in the Weddell Sea (> 55°S, 10°–60°W, n = 6); and 68 ± 8% and 60 ± 9%, respectively, in winter water in the Ross Sea (> 55°S, 150°–180°W or 175°–180°E, n = 26). These saturation percentages were within the range of saturations for DSW determined in this study. Similar to the LCDW, the pCFC-12 and pSF_6_ of the DSW were assumed to be at the constant ratios to the atmospheric partial pressures (gray line in Fig. 4). The pCFC-12 and pSF_6_ in the AABW formed during a given year should be distributed along the line connecting the LCDW (blue line in Fig. 4) with the DSW (gray line in Fig. 4) during that year (red dashed lines in Fig. 4). This temporal varying linear relationship connecting the LCDW with the DSW was compared with observations to determine the year when the observed AABW was formed. Finally, we quantified each age using the difference between the estimated formation year and the observed year. By applying this method, the age of the AABW can be quantified regardless of the mixing ratio of the LCDW and the DSW. In addition, the age of AABW was determined not by the saturation values of pCFC-12 and pSF_6_ in DSW, but by the ratio of saturations of these gases. This method is similar to pSF_6_/pCFC-12 ratio age, excluding the aspect of considering saturation degrees of the gases in LCDW and DSW.

The uncertainty in the water mass age of the AABW is primarily caused by the assumption of the DSW property. We therefore checked the sensitivity of the water mass age by changing the pCFC-12 and pSF_6_ of the DSW. When the pCFC-12 of DSW was set to 94% (78 + SD) and 62% (78 − SD) of the atmospheric partial pressure, the age of the bottom water mass changed by − 2 years and 4 years, respectively. When the pSF_6_ of the DSW was set to 75% (62 + SD) and 49% (62 − SD) of the atmospheric partial pressure, the ages changed by 3 years and − 2 years, respectively. The other errors in the age of the AABW can arise from uncertainties in measurements and the reconstruction of pCFC-12 and pSF_6_ in LCDW. The former (± 2% in pCFC-12 and ± 7% in pSF_6_) led to ± 2 years in AABW age. For the latter, when the pCFC-12 and pSF_6_ of LCDW were set to zero, AABW age changed by 2 years. The spatial and vertical distributions of the AABW ages were mostly unchanged by these pCFC-12 and pSF_6_ uncertainties.

### Applicability of the estimation method in an ocean model

The observed pCFC-12 and pSF_6_ data in the region off CD are limited in space and time. Therefore, it is necessary to check whether the estimation method of AABW age, with the assumption that pCFC-12 and pSF_6_ in AABW can be explained by the mixing of two endmembers (DSW and LCDW) for each formation year, can be applied in this region regardless of time. Here, to investigate the applicability of the estimation method, we conducted a numerical simulation of an ocean–sea ice–ice shelf model that included CFC-12 and SF_6_ in the Southern Ocean. The configuration of the ocean–sea ice–ice shelf model was the same as that in ref.^45^, but with different surface forcing^46^. In this study, we utilized present-day daily climatological atmospheric forcing throughout the simulation. This model with this surface forcing can realistically reproduce coastal sea ice production along the Antarctic coastal margins compared to satellite-based estimations^45,47^. The vertical resolution in this model is much finer than that in a typical z-coordinate model and thus it is intended to better represent the water mass exchange across the shelf break regions^45^. The implementation of CFC-12 and SF_6_ in the model followed that of the Ocean Model Intercomparison Project biogeochemical (OMIP-BGC) protocols^48^. After 30 years of spin-up integration of physical variables from an initial rest state, we obtained a quasi-steady state in the model and then performed a numerical simulation with CFC-12 and SF_6_ from 1940 to 2020. The atmospheric concentrations of CFC-12 and SF_6_ were constant in the domain, but varied year-to-year, using the same atmospheric data described in the previous subsection.

Here, we focused on the simulated results in the region off CD. Figure 5a–c show the relationships between the simulated pCFC-12 and pSF_6_ in new AABW (in the region near the CD polynya; 63°–67°S, 60°–68°E, *θ* <  − 0.4°C^10^). Similar to the observed new AABW, the simulated pCFC-12 and pSF_6_ in the new AABW were linearly distributed (R > 0.99, p < 0.01; Fig. 5a). In addition, the simulated saturations of pCFC-12 and pSF_6_ in new AABW (pCFC-12: 40%, pSF_6_: 35%; mean from 1980 to 2020) were close to those in the observed new AABW (pCFC-12: 38 ± 8%, pSF_6_: 30 ± 7%; green asterisks in Fig. 4). This indicates that the model reasonably reproduces the mixing ratio of DSW and LCDW during the AABW formation. The simulated saturations of pCFC-12 and pSF_6_ in DSW in CD polynya were 68% and 63%, respectively (mean from 1980 to 2020; gray lines in Fig. 5a,b), which were within the range of saturations for those estimated from observed data in the previous subsection. Such consistency with the observational results gives us confidence in the model results.

Using this model, we checked the applicability of the estimation method. After 1980, the slope of the relationship between pCFC-12 and pSF_6_ in new AABW reflected the atmospheric pSF_6_/pCFC-12 ratio of the formation year (e.g., Slope =  ~ 0.013 in 2010 and ~ 0.020 in 2020; Fig. 5a,c). This means that the linear relationship between pCFC-12 and pSF_6_ of new AABW varies year-to-year in response to the concentrations in DSW, which are closely related to the atmospheric partial pressures in the corresponding year. As shown in Fig. 5b, higher levels of pCFC-12 and pSF_6_ were observed in colder AABWs. The model results indicate that pCFC-12 and pSF_6_ in new AABW off CD can be explained by the mixing of the two endmembers: old, warm LCDW (almost zero pCFC-12 and pSF_6_) and cold DSW (temporally varying pCFC-12 and pSF_6_). These results support the water mass age estimation method described in the previous subsection, assuming that the relationship between pCFC-12 and pSF_6_ in the AABW reflects mixing of LCDW and DSW during the year when the AABW was formed.

### Spatial and vertical AABW age distributions

**Figure 6:**
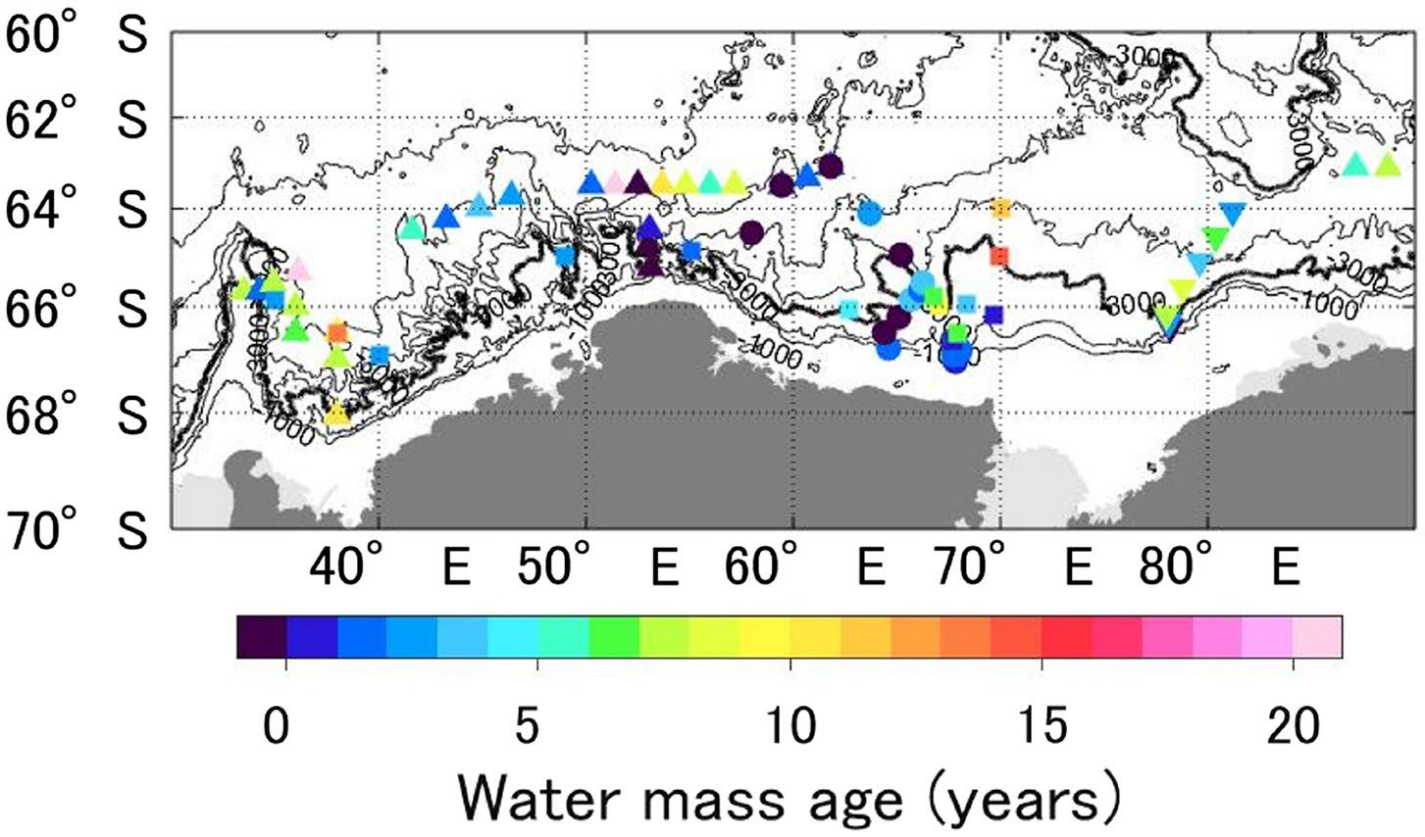
Spatial distribution of bottom AABW ages (neutral density: *γ*^n^ > 28.27 kg m^−3^) (mean of observations within 100 m of the bottom). The symbols used are the same as in Fig. 1. Black contour lines represent isobaths at 1000 m intervals from 1000 to 3000 m and at 500 m intervals below 3000 m. The 3000 m isobath is indicated by the thick black line. The figure was generated using MATLAB (version R2021a; https://www.mathworks.com/products/matlab.html) and the M_Map toolbox (http://www.eoas.ubc.ca/~rich/map.html).

Figure 6 shows distribution of the AABW ages estimated from the pCFC-12 and pSF_6_. Along the western canyons off CD and along the ~ 3000 to 3500 m isobaths (except at 65.8°–66.5°S in Wild Canyon), the bottom water mass age was < 5 years, which was younger than those in the east and in the northwestern offshore of CD. This distribution of new AABW is roughly consistent with the suggested pathway of new CDBW^10^. Given near-bottom velocities of ~ 0.1 m s^−1^ (ref.^10^) and ~ 0.05 m s^−1^ (ref.^49^), new CDBW takes several months to traverse the Wild/Daly Canyons, and a few years to reach ~ 40°E along the isobath, respectively. Although this estimate is rough or underestimation because the velocity observations are limited and the water masses generally do not move in a straight line, the estimated water mass age of new AABW in this study is roughly consistent with the timescale of new CDBW spread estimated from the velocity data. The spatial distribution of AABW ages in Fig. 6 substantially describes the detailed pathways and timescale of the spread of CDBW.

The older AABWs were found in the east of CD (up to 8 years) and in the northwestern part of the study area (up to > 20 years) (Fig. 6). These AABWs were also high in salinity (Fig. 3b). The temperature was, however, higher in the east and lower in the northwest (Fig. 3a). According to the characteristics of the different AABW types described in ref.^14^, the old AABW on the eastern side with higher salinity and warmer temperature reflects the inflow through the PET, while saline but cold AABW in the northwestern part of the study area is the AABW from the Weddell Sea (WSDW; refs.^14,16^). The water mass ages estimated in this study indicate that the WSDW in the study area is older than the AABW that flows through the PET. This seems reasonable considering the fact that the distance from the formation region to the study area for the WSDW (Weddell Sea) is longer for the AABW through the PET (Adélie Land coast and Ross Sea), although the velocities and transit times of these AABW types have not been clarified in detail. Note that the age of these waters can be reliable if they are directly advected from the source region or mixed only with LCDW that contains negligible or no tracers. AABW far from the source region can be significantly affected by mixing with older and/or different AABWs along the spreading pathway. The mixture should have mean pCFC-12 and pSF_6_ values but not the mean ratio (and age) as ratios do not mix linearly. This could introduce an error in our age estimation. Furthermore, WSDW would contain older CDBW, advected westward into the Weddell Sea, where it mixes with the newly formed AABW in the Weddell Sea (e.g., ref.^10^). Similarly, AABW through the PET is a mixture of Adélie Land Bottom Water and older Ross Sea Bottom Water^3,15^. These facts complicate the age estimation of these AABWs. To investigate how tracer concentrations and age in AABW far from the source region change along the spreading pathway, tracer observations over a wider area along the spreading pathway are required.

**Figure 7:**
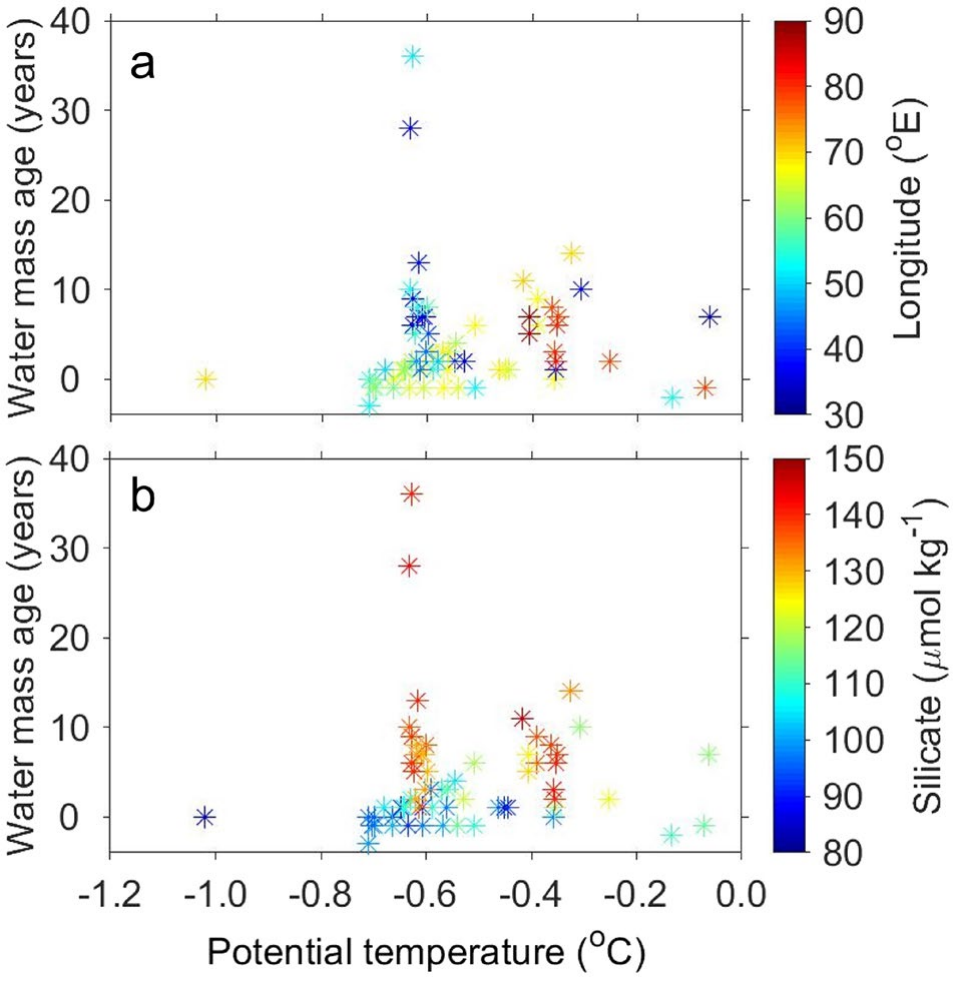
Relationship between potential temperature and water mass age of bottom AABW (neutral density: *γ*^n^ > 28.27 kg m^−3^) (mean of observations within 100 m of the bottom). The data used are the same as in Fig. 6. The colors denote (**a**) longitude and (**b**) silicate concentration.

Temperature has been used to trace newly formed AABW from its source region (e.g., ref.^3^). However, the relationship between the potential temperature and water mass age of AABW was not correlated in the study area (R = 0.05, p > 0.68; Fig. 7), i.e., cold AABW did not necessarily correspond to new AABW. For example, waters with temperatures of − 0.7 °C to − 0.6 °C exhibit a broad range of ages (− 1 to 36 years). As described above in this subsection, the old AABW (> 20 years) in the west of 52°E and the new AABW in the east of 60°E reflect the spread of WSDW and CDBW, respectively, although both these old and new AABWs are characterized by temperatures of − 0.7 °C to − 0.6 °C. The results of this study highlight the importance of measuring the concentrations of CFC-12 and SF_6_ to clearly distinguish the AABW that flows in from different source regions. Oxygen concentrations, converted to apparent oxygen utilization (AOU: the difference between the saturation and observed dissolved oxygen concentrations) were also similar (~ 115 µmol kg^−1^) between AABWs at this temperature range (not shown). Silicate concentrations, however, were different between old and young AABWs; high (> 130 µmol kg^−1^) in old AABW and low (< 110 µmol kg^−1^) in new AABW (Fig. 7b). This result supports that CFC-12 and SF_6_ can successfully distinguish new and old AABW formed in different source regions.

**Figure 8:**
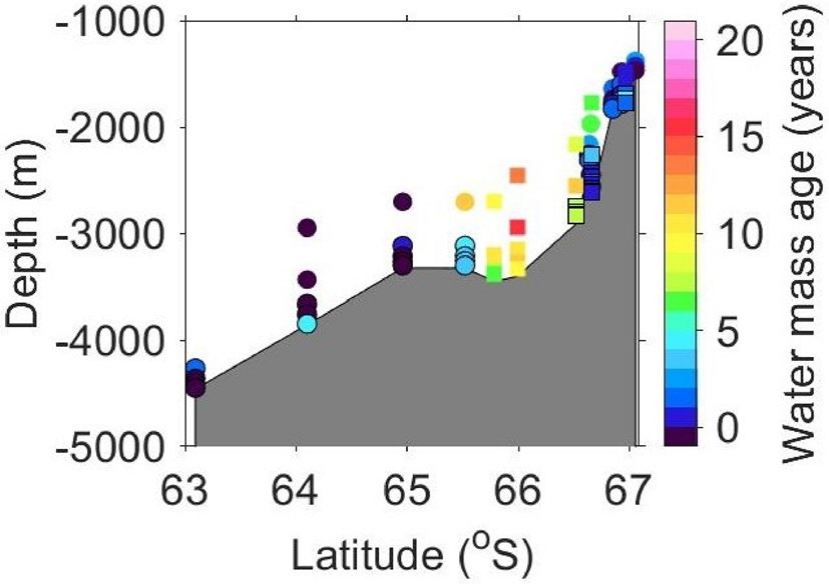
Vertical distribution of AABW ages (neutral density: *γ*^n^ > 28.27 kg m^−3^) along Wild Canyon (white line in Fig. 1). Data were obtained during cruises of KH19-01 (circles) and KH20-01 (squares). The symbols of new AABW (*θ* <  − 0.4 °C)^10^ are outlined in black. The figure was generated using MATLAB (version R2021a; https://www.mathworks.com/products/matlab.html).

The distribution of the water mass age of AABW along Wild Canyon was investigated in more detail (Fig. 8). Near the bottom, the water mass age of AABW was younger than that of the shallower layer, indicating that the new CDBW descended along the bottom of the canyon. At 65.8°–66.5°S in 2020, the bottom water mass age was relatively old (~ 6–10 years). These waters also had higher temperature and higher salinity (*θ* >  − 0.4 °C, *S* > 34.65 at 65.8°S and 66.0°S; *θ* =  − 0.5 °C, *S* = 34.64 at 66.5°S; Fig. 2a,b) compared to younger waters at nearby stations (*θ* <  − 0.6 °C, *S* < 34.61). These *θ*–*S* properties are close to that of waters in the east of CD (Fig. 3a,b). Thus, it is suggested that the AABW at 65.8°–66.5°S in Wild Canyon in 2020 is influenced by the older AABW inflowing through the PET. According to refs.^10,50^, CDBW outflow occurs sporadically. In the Wild Canyon near the source region of DSW, the water property would change between those of new CDBW and older AABW inflowing through the PET on a short timescale. The older AABW at 65.8°–66.5°S might reflect such short timescale variability. Otherwise, locations of these stations might be slightly off the pathway of new CDBW along the canyon. Another possible explanation is that CDBW production was lower in the winter of 2019 than in 2018 since sea ice production in the CD polynya was significantly lower in the winter of 2019, according to the calculation by ref.^51^.

## Summary and conclusions

We analyzed CFC-12 and SF_6_ in the region off CD to quantitatively understand the pathways and timescale of the spread of AABW. The pCFC-12 and pSF_6_ of the LCDW/DSW were estimated to be 9%/78% and 6%/62% of their atmospheric partial pressures, respectively. We found that based on the temporally variable linear relationship between pCFC-12 and pSF_6_ in the AABW that reflects the mixing of LCDW and DSW during the year of AABW formation, the water mass age of AABW can be estimated. A numerical simulation of CFC-12 and SF_6_ using a coupled ocean–sea ice–ice shelf model confirmed that the estimation method is suitable for application.

The AABW ages were estimated using the pSF_6_/pCFC-12 ratio, which considers the saturation degrees of the gases in LCDW and DSW. Along the western canyons off CD and along the ~ 3000 to 3500 m isobaths, the bottom age of the AABW was only < 5 years, reflecting the spread of new CDBW. Older ages of up to ~ 8 years and > 20 years were obtained for the AABW through the PET in the east of CD and for the WSDW in the northwestern region of the study area, respectively. This study revealed the age distribution of CDBW as well as AABW formed in other regions. The age estimation method in this study is particularly useful for studying the AABW in the southern part of the Southern Ocean, where previous methods (e.g., TTD and pSF_6_ age) cannot be applied (e.g., refs.^18,27^). Knowing the age and the spread timescale of AABW should be useful for understanding biogeochemical cycles at the bottom, such as the oxygen consumption rate and the nutrient regeneration rate. Furthermore, using this age estimation method for AABW, it is possible to detect and quantify the changes in AABW formation and spread under the ongoing freshening and warming of Antarctic waters.

## Methods

### Study area and data

This study focused on the spread of AABW in the Indian Ocean sector of the Southern Ocean (30°–90°E), including off CD (Fig. 1). We used hydrographic observational data in 2013, 2016, 2019, and 2020 (cruises of MR12-05, WHP I08S, KH19-01, and KH20-01, respectively; see Supplementary Table S1 for details). Data in 2013 (MR12-05)^52^ and 2016 (WHP I08S)^53^ were obtained from the CLIVAR Carbon Hydrographic Data Office website (https://cchdo.ucsd.edu/). For more details on these observations, please refer to the corresponding references. Data in 2019 and 2020 were obtained during R/V Hakuho-maru cruises. Temperature, salinity, and pressure were measured using a conductivity-temperature-depth (CTD) profiler (Sea-Bird Electronics 9plus). The CTD salinity was calibrated using the water sample salinities measured with a salinometer (Guildline AUTOSAL 8400B), according to the method of World Ocean Circulation Experiment standard. Silicate concentrations in the water samples were measured using a QuAAtro system according to the method of ref.^54^. Certified reference materials (CRM) for nutrients provided by KANSO Co. Ltd. were used for each run. Water samples for CFC-12 and SF_6_ analyses were collected into 300 ml glass bottles. Sampled water was transferred to a purge and trap system and analyzed using an electron capture detector-gas chromatography system based on the method described in ref.^55^. The precisions of the analyses were 2% or 0.02 pmol kg^−1^ for CFC-12 and 7% or 0.05 fmol kg^−1^ for SF_6_. The concentrations of CFC-12 and SF_6_ in each water sample were converted to partial pressures (pCFC-12 and pSF_6_: ppt) to remove their dependence on temperature and salinity^56–58^.

## Supplementary Information

Supplementary Table S1.

## Data Availability

MATLAB (version R2021a; https://www.mathworks.com/products/matlab.html) was used to analyze the data. The datasets generated and analyzed during the current study are available from the corresponding author on reasonable request.
