# Supplementary material for: Age distribution of Antarctic Bottom Water off Cape Darnley, East Antarctica, estimated using chlorofluorocarbon and sulfur hexafluoride
Source: Sci Rep. 2022 May 19;12:8462. doi: 10.1038/s41598-022-12109-4 (PMC9120186; doi:10.1038/s41598-022-12109-4)
Supplement: Supplementary file 1 — Supplementary Table S1. [file 41598_2022_12109_MOESM1_ESM.pdf]

Supplementary Information

Age distribution of Antarctic Bottom Water off Cape Darnley, East Antarctica, estimated using chlorofluorocarbon and sulfur hexafluoride

Yoshihiko Ohashi[1,*], Michiyo Yamamoto-Kawai[1], Kazuya Kusahara[2], Ken'ichi Sasaki[3], and Kay I. Ohshima[4,5]

[1]Department of Ocean Sciences, Tokyo University of Marine Science and Technology, Tokyo, 108-8477, Japan

[2]Research Center for Environmental Modeling and Application, Research Institute for Global Change, Japan Agency for Marine-Earth Science and Technology, Yokohama, 236-0001, Japan

[3]Mutsu Institute for Oceanography, Research Institute for Global Change, Japan Agency for Marine-Earth Science and Technology, Mutsu, 035-0022, Japan

[4]Institute of Low Temperature Science, Hokkaido University, Sapporo, 060-0819, Japan

[5]Arctic Research Center, Hokkaido University, Sapporo, 001-0021, Japan

[*]Corresponding author, email: yohash0@kaiyodai.ac.jp

The file contains:

Supplementary Table S1

Supplementary Table S1. Observational data used in this study.

Cruise name	Period	Platform	Reference
MR12-05	Jan.–Feb. 2013	Mirai	Uchida et al. (2015) ref.[52]
WHP I08S	Feb.–Mar. 2016	Roger Revelle	Macdonald (2016) ref.[53]
KH19-01	Jan.–Feb. 2019	Hakuho-maru	*
KH20-01	Jan.–Feb. 2020	Hakuho-maru	*

*The data was used for the first time in this study.
